# Renal proximal tubule cells: power and finesse

**DOI:** 10.1172/JCI169607

**Published:** 2023-05-01

**Authors:** Michaela A.A. Fuchs, Myles Wolf

**Affiliations:** 1 Division of Nephrology, Department of Medicine, and; 2 Duke Clinical Research Institute, Duke University School of Medicine, Durham, North Carolina, USA.

## Abstract

The proximal tubule is the high-capacity reabsorptive powerhouse of the kidney. Two papers in recent issues of the *JCI* highlight mechanisms of more delicate effects of the proximal tubule. Yoon et al. demonstrated the intracellular mechanism by which parathyroid hormone (PTH) increases production of 1,25-vitamin D. Activation of PTH receptor 1/cAMP/PKA signaling inhibited salt-inducible kinase 2 (SIK2) and SIK3, which increased *CYB27B1* transcription and 1,25-vitamin D production. Replication of these effects with small-molecule SIK inhibitors suggests possible therapeutic applications for patients with disorders characterized by 1,25-vitamin D deficiency. Zhou et al. discovered that proximal tubule glycolysis acts as a phosphate sensor that regulates fibroblast growth factor 23 production in bone. They described several kidney-specific metabolic modifications that enabled glycolysis to be deployed as a phosphate sensor. The provocative results raise intriguing questions with implications for patients with disorders of phosphate homeostasis, including chronic kidney disease.

## The proximal tubule

In healthy adults, the kidneys filter more than 150 liters of blood daily, which is 30–50 times the total blood volume. This prolific redundancy ensures efficient excretion of water-soluble waste products and any salt, water, and minerals that individuals ingest in excess of physiological need. It also explains why kidney donors and kidney transplant recipients live well with just one kidney and why early chronic kidney disease (CKD) can be notoriously difficult to diagnose.

The remarkably sophisticated renal tubule backstops the torrent of filtration. It must reabsorb more than 99% of the glomerular filtrate while tightly regulating circulating concentrations of electrolytes and minerals and maintaining water and acid-base balance. The workhorse of the tubule is the proximal tubular epithelium, which typically reabsorbs more than 100 liters of glomerular filtrate daily. The hyperactive, energy-consuming, proximal tubule epithelial cells are powered by an abundance of mitochondria, which makes them the main target of ischemic acute kidney injury. Two innovative studies in recent issues of the *JCI* center even more attention on the proximal tubule, focusing less on its well-known brawn and more on its elegant contributions to systemic calcium and phosphate homoeostasis.

## Calcium sensing and regulation by PTH

Parathyroid hormone (PTH) regulates serum calcium minute to minute based on continuous feedback from the calcium-sensing receptor, which is a G protein–coupled receptor that is highly expressed in the parathyroid gland (1). When hypocalcemia is sensed, resultant increases in PTH stimulate calcium (and phosphate) release from bone, augment calcium reabsorption in the distal tubule, and increase circulating concentrations of 1,25-vitamin D by increasing CYP27B1 expression and decreasing CYP24A1 expression in the proximal tubule. PTH also stimulates phosphaturia, which ensures excretion of the phosphate released from bone into circulation in response to PTH. Recent studies shed light on the genomic regulation of *CYP27B1* and *CYP24A1* (2, 3), but the intracellular pathways that exert these effects downstream of PTH receptor 1 binding have been unclear.

**Figure 1:**
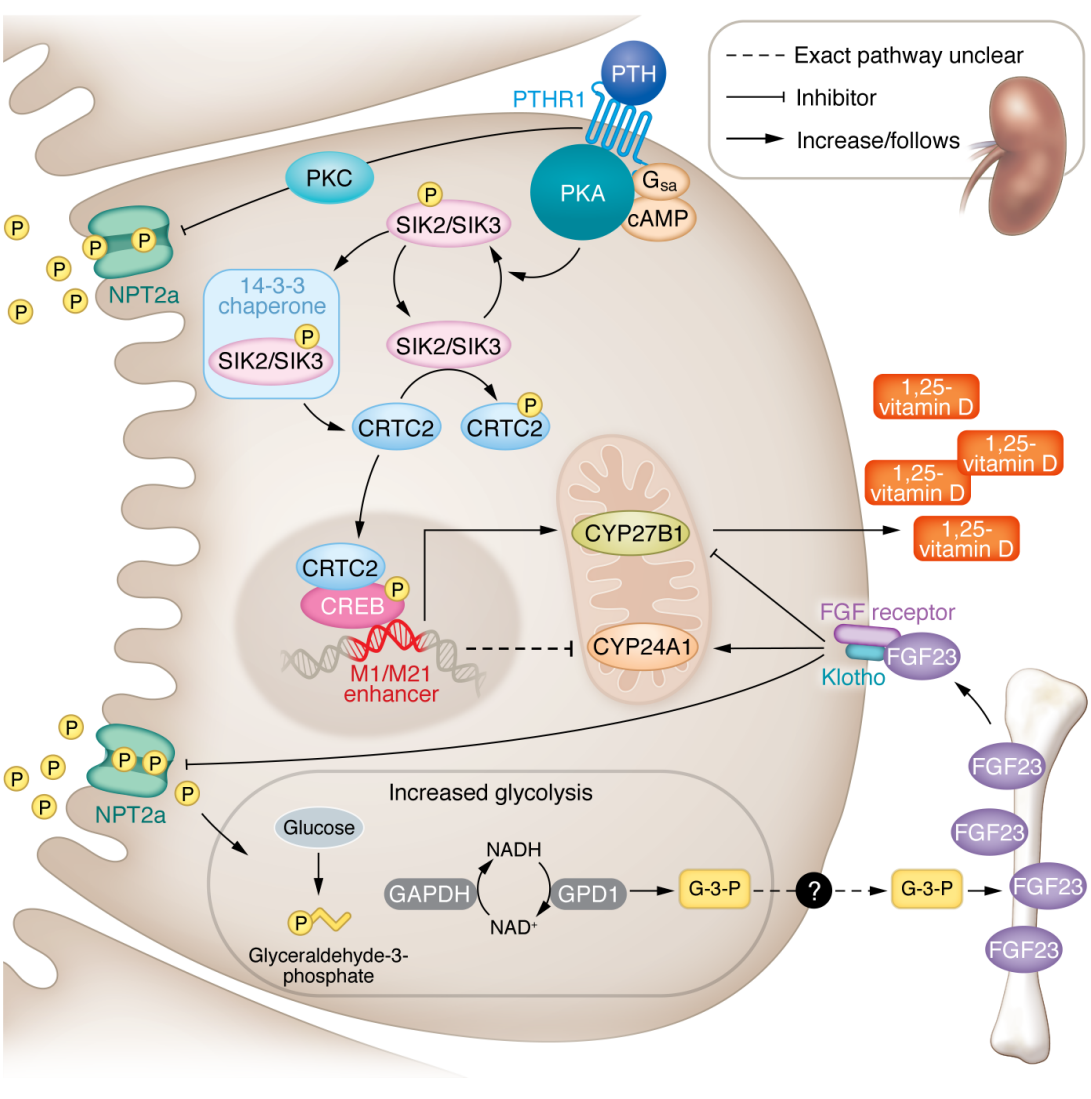
SIK2/SIK3 regulates 1,25-vitamin D production, and glycolysis acts as a phosphate sensor in proximal tubular epithelia. PTH binding to its receptor drives cAMP-mediated PKA phosphorylation of SIK2/SIK3. Phosphorylation of SIK2/SIK3 leads to binding of 14-3-3 chaperone proteins that inhibit the constitutive phosphorylation of SIK targets. Inhibition of SIK-mediated phosphorylation liberates cofactor CRTC2 and facilitates its translocation into the nucleus. Nuclear binding of CRTC2 to pCREB at the M1/M21 enhancer leads to increased transcription of *CYP27B1* and, via an incompletely understood mechanism, downregulation of *CYP24A1*. Increased CYP27B1 and decreased CYP24A1 activity in response to PTH or SIK inhibition increases circulating 1,25-vitamin D levels. Through an effect of PTH that is independent of SIK2/SIK3, PTH receptor 1 (PTHR1) activation also reduces phosphate reabsorption via PKC-mediated phosphorylation of scaffolding proteins, which leads to downregulation of NPT2a function in the apical membrane. NPT2a is the entrance pathway for inorganic phosphate that serves as the rate-limiting substrate for GAPDH-catalyzed glycolysis in proximal tubules. Cytosolic NAD^+^ recycling by GPD1 produces G-3-P, which is released into the circulation through an unknown mechanism and stimulates FGF23 production in bone. Closing the feedback loop, binding of FGF23 to FGF receptor–klotho coreceptor complexes leads to downregulation of NPT2a function and increased renal phosphate excretion. Opposing the analogous effects of PTH, FGF23 also inhibits CYP27B1 and stimulates CYP24A1 activity.

In a recent issue of the *JCI*, Yoon et al. (4) used rigorous pharmacologic and genetic tools to demonstrate in mice and in human kidney organoids that PTH increased proximal tubular production of 1,25-vitamin D by inhibiting salt-inducible kinase 2 (SIK2) and SIK3 (Figure 1). Inhibition of SIK2/SIK3 by PTH increased translocation of cofactor CRTC2 into the nucleus, which bound phosphorylated cAMP response element-binding protein (pCREB) and activated the previously identified M1/M21 enhancers of *CYP27B1* (3, 4). The authors were able to replicate these effects of PTH using small-molecule SIK inhibitors, which increased circulating concentrations of 1,25-vitamin D and calcium and suppressed PTH. These results build on the group’s previous work on the intracellular mechanism of PTH actions in osteocytes and their ChIP-Seq data (3, 5). Interestingly, SIK inhibition exerted no direct effect on renal phosphate handling, which indicates that distinct pathways mediate different actions of PTH in the proximal tubule.

The authors speculated that SIK inhibition could be a therapeutic paradigm for CKD, based on their observation that SIK inhibition increased 1,25-vitamin D levels in mice with CKD despite increased systemic FGF23 levels. However, this conclusion was based on just four hours of observation after a single injection of the SIK inhibitor in a specific mouse model of CKD characterized by intact tubular function (6). Based on the authors’ data in mice with genetic deletion of SIKs, longer durations of SIK inhibition would be expected to increase 1,25-vitamin D levels while also increasing serum calcium and FGF23, suppressing PTH and potentially causing adverse effects on bone. It is unclear what would be the clinical goal of SIK inhibition in patients with CKD and whether there would be a sufficient therapeutic window within which potential benefits would outweigh risks.

Alternatively, SIK inhibition could be used to treat primary hypoparathyroidism, especially etiologies that are unlikely to benefit from calcilytics, such as surgical parathyroidectomy (7). Bypassing absent PTH to restore its end-organ effects could raise 1,25-vitamin D and calcium levels and improve bone health by restoring anabolic effects of PTH on bone. Although SIK inhibition is not involved in PTH-mediated phosphaturia, indirectly, SIK inhibition might even correct hyperphosphatemia in hypoparathyroidism, which is a consequence of absent PTH and inappropriately low FGF23. By increasing 1,25-vitamin D and calcium levels, SIK inhibition would likely restore normal FGF23 secretion and, thus, correct hyperphosphatemia, as seen in patients with hypoparathyroidism who are treated with calcitriol or calcium (8, 9).

Nevertheless, the finding that SIK inhibition can increase 1,25-vitamin D levels in CKD despite high FGF23 levels is fascinating. It is interesting to speculate whether this result might indicate crosstalk between FGF23-mediated inhibition of CYP27B1 and SIK inhibitor–mediated stimulation of CYP27B1. At first glance, crosstalk seems unlikely because FGF23-FGF receptor binding activates ERK signaling, whereas SIK phosphorylation is cAMP/PKA dependent. However, FGF21 binding to FGF receptors regulates other AMP-activated kinases via interactions between ERK1/2 and liver kinase B1, which is also an upstream regulator of SIKs (10). Further studies are needed to investigate the mechanism of how SIK inhibitors enable CYP27B1 to escape inhibition by FGF23.

## Phosphate sensing and regulation by FGF23

Unicellular organisms sense extracellular phosphate directly via transmembrane proteins (11). The quest to identify a specific protein akin to the calcium-sensing receptor as the mammalian phosphate sensor has been futile to date. In another paper in the *JCI*, Zhou et al. (12) discover that proximal tubular glycolysis — a metabolic pathway, not a discrete protein — acts as a phosphate sensor in the kidney.

Unlike other cells in which glucose is the rate-limiting substrate for glycolysis, Zhou et al. demonstrated that phosphate was uniquely rate limiting in the proximal tubule. It is also unique that the source of phosphate was neither intracellular nor circulating, but rather phosphate that was reabsorbed from the glomerular filtrate via sodium phosphate cotransporter 2a (NPT2a). GAPDH, which catalyzes the phosphate-dependent step in glycolysis, was coupled with increased production of glycerol-3-phosphate (G-3-P) from dihydroxyacetone-phosphate (DHAP) by cytosolic G-3-P dehydrogenase 1 (GPD1). This step replenished NAD^+^, which was needed by GAPDH to maintain glycolysis. Importantly, phosphate-induced increases in glycolytic flux, leading to increased G-3-P production, was a feature specific to the kidney that was not observed in other tissues such as heart, liver, and skeletal muscle, which instead rely on lactate dehydrogenase (LDH) to replenish NAD^+^ for continued glycolysis. Once again, unlike other cells that transport G-3-P into the mitochondria to reform DHAP by GPD2, G-3-P was secreted via an unknown pathway into the circulation where it stimulated FGF23 production in bone. In turn, increased FGF23 completed the feedback loop by downregulating proximal tubular NPT2a, thereby reducing phosphate uptake, glycolytic flux, and thus, G-3-P production (Figure 1).

The finding that NAD^+^ recycling by GPD1 lies at the core of phosphate sensing is striking for many reasons. First, proximal tubule cells are laden with mitochondria that can regenerate NAD^+^ while synthesizing ATP, yet it is the less energy efficient regeneration of the cytosolic NAD^+^ pool that played a prominent role in the proposed mechanism. Second, GPD1 overshadowed LDH-catalyzed conversion of pyruvate to lactate, which usually is the preferred mechanism of cytosolic regeneration of NAD^+^. Perhaps favoring GPD1 over LDH helps the proximal tubule compartmentalize phosphate sensing from acid-base regulation that could be altered by undulating lactic acid production. Regardless, the mechanism by which activation of glycolysis led to flux through GPD1 in proximal tubule cells, but not other cell types, remains to be identified. Third, loss of carbon equivalents via extracellular G-3-P secretion in lieu of completion of the glycerol-phosphate shuttle to produce DHAP and FADH_2_ in the mitochondria seems counterproductive in energy-intensive cells. Perhaps this process explains, in part, the primacy of GPD1 over LDH for NAD^+^ regeneration. An energy-intensive cell that sheds carbon equivalents for the purpose of phosphate homeostasis cannot afford to convert pyruvate to lactate instead of shuttling it to the mitochondria to form acetyl-CoA. Collectively, these findings indicate that the proximal tubule co-opts a fundamental and ubiquitous metabolic pathway — glycolysis — and converts it into a highly specialized transducer of the signal from kidney to bone that regulates phosphate homeostasis.

Great studies answer critical questions and simultaneously prompt numerous exciting new ones. Zhou et al. (12) solved the previously vexing question of how the bone seems to know to make more FGF23 in response to dietary-phosphate loading without an intermediate change in serum phosphate (13). The authors also address the previous paradox that FGF23 regulates serum phosphate, but increases in serum phosphate itself do not directly stimulate FGF23 production. Indeed, other investigators use calcium, or 1,25-vitamin D, but not phosphate, to stimulate FGF23 expression in vitro (14). However, the Zhou et al. study (12) did not address whether the proximal tubular G-3-P response was dependent on the phosphate concentration in the tubular lumen or the absolute amount of phosphate entering the proximal tubular cell. The human experiment reported by Zhou et al. involved an enormous 1.5-gram oral bolus of phosphate (and the mouse studies used large intravenous doses) that briefly increased the serum phosphate and, thus, the phosphate concentration in the glomerular filtrate (12). More typical 500 mg phosphate meals do not increase serum phosphate (13). If an increase in the absolute amount of reabsorbed phosphate is sufficient to drive G-3-P production, do stimuli that increase glomerular filtration and, thus, the amount of phosphate that must be reabsorbed, also increase G-3-P and FGF23? Furthermore, what happens during the fasting state and in patients with diabetes when a shift from glycolysis to gluconeogenesis reduces G-3-P production — do other stimuli intervene to regulate FGF23 (15, 16)? Or might the duration of G-3-P–mediated stimulation of FGF23 extend into the fasting state beyond the initial surge of G-3-P? The minimal diurnal variation in FGF23 levels despite periods of feeding and fasting supports differential durations of the G-3-P and FGF23 responses (17), but further study is needed.

The mechanism of the differential effects of G-3-P versus other stimuli on FGF23 regulation also warrants further investigation. Iron deficiency and inflammation stimulate FGF23 transcription in osteocytes and simultaneously increase intracellular cleavage of FGF23 (14, 18, 19). This mechanism results in elevated circulating concentrations of FGF23 fragments but normal levels of full-length, biologically active FGF23 and, thus, normal serum phosphate. In contrast, G-3-P drives increases in full-length FGF23, which lowers serum phosphate. The increase in full-length FGF23 suggests that G-3-P might coordinate pathways in which FGF23 production is upregulated while cleavage is simultaneously downregulated. This coordination could occur by modulating the degree of posttranslational glycosylation versus phosphorylation of FGF23 that determines whether it is cleaved or secreted intact (20). Alternatively, perhaps regulation of FGF23 cleavage differs across cells that respond to G-3-P, namely osteocytes versus bone marrow stromal cells.

Zhou et al. (12) build on a prior discovery by their research team related to increased renal production of G-3-P as the kidney’s distress signal to inform the bone to increase FGF23 levels in ischemic acute kidney injury (21). In this setting, recycling of NAD^+^ in the cytosol, by GPD1 and LDH, becomes absolutely essential because oxidative phosphorylation is impaired. In contrast, how might the mechanism of phosphate sensing described by Zhou et al. (12) map to CKD, in which NPT2a and proximal tubular phosphate reabsorption are downregulated and FGF23 levels are constitutively elevated? Perhaps the switch from β-oxidation of fatty acids to glycolysis as the proximal tubule’s primary energy source in CKD (22) results in sufficient ongoing G-3-P production to continuously stimulate FGF23 production. If so, the phosphate-sensing apparatus could become maladaptive in CKD if chronically exporting carbon in the form of G-3-P further depletes the cells of energy and promotes CKD progression. This hypothesis raises the intriguing possibility that inhibitors of sodium-glucose transporter 2 may limit proximal tubule glucose availability and, thereby, decrease G-3-P production and efflux as one of the elusive mechanisms through which these drugs protect against CKD progression (23).

In summary, Yoon et al. (4) and Zhou et al. (12) remind us that proximal tubular cells must be recognized not only for their power as the kidney’s reabsorptive workhorse cells, but also for their extraordinary endocrine finesse in regulating vitamin D and phosphate homeostasis.

## Version 1 05/01/2023

Electronic publication
